# *Suaeda australis* and its associated rhizosphere microbiota: a comparison of the nutrient removal potential between different shrimp farm sediments in New Caledonia

**DOI:** 10.3389/fmicb.2023.1260585

**Published:** 2023-10-09

**Authors:** Marie Colette, Linda Guentas, Luc Della Patrona, Dominique Ansquer, Nolwenn Callac

**Affiliations:** ^1^French Institute for Research in the Science of the Sea (IFREMER), Research Institute for Development (IRD), University of New Caledonia, University of Reunion, CNRS, UMR 9220 ENTROPIE, Nouméa, New Caledonia; ^2^Institute of Exact and Applied Sciences (ISEA), University of New Caledonia, Nouméa, New Caledonia

**Keywords:** halophyte, earthen pond sediment, active rhizosphere microbiota, nutrient removal, metabarcoding

## Abstract

Shrimp rearing generate organic waste that is trapped in the pond sediment. In excess, these wastes may impair aquaculture ecosystem and shrimps’ health. To promote the biological oxidation of accumulated organic waste, the pond is drained and dried at the end of each production cycle. However, this practice is not always conducive to maintaining microbial decomposition activities in sediments. Shrimp production in New Caledonia is no exception to this problem of pollution of pond bottoms. One promising way of treating this waste would be bioremediation, using a native halophyte plant and its microbiota. Thus, this study explored the nutrient removal potential of *Suaeda australis* and its microbiota on sediments from four shrimp farms. *Suaeda australis* was grown in an experimental greenhouse for 6 months. In order to mimic the drying out of the sediments, pots containing only sediments were left to dry in the open air without halophytes. An analysis of the chemical composition and active microbiota was carried out initially and after 6 months in the sediments of the halophyte cultures and in the dry sediments for each farm, respectively. In the initial state, the chemical parameters and the microbial diversity of the sediment varied considerably from one farm to another. Growing *Suaeda australis* reduced the nitrogen, phosphorus and sulfur content in all type of sediment. However, this reduction varied significantly from one sediment to another. The rhizosphere of *Suaeda australis* is mainly composed of micro-organisms belonging to the *Alphaproteobacteria* class. However, the families recruited from this class vary depending on the farm in question. Depending on the sediment, the variation in microbiota leads to different putative biochemical functions. For two of the farms, a similar reduction in nitrogen concentration was observed in both dry and cultivated sediments. This suggests that certain initial chemical characteristics of the sediments influence the nutrient removal efficiency of *Suaeda australis*. Our study therefore highlights the need to control the pH of sediments before cultivation or in dry sediments in order to ensure optimal microbial decomposition of organic waste and nutrient cycling.

## 1. Introduction

In New Caledonia, the Pacific blue shrimp *Penaeus stylirostris* is farmed mainly on a semi-intensive basis. On this South Pacific island, the shrimps are farmed in earthen basins of between 3 and 12 hectares dug directly into the saltpans (Goarant et al., 2004; Della Patrona and Brun, 2009). During the rearing, the uneaten feed but also feces, shrimp’s exoskeleton, and dead phytoplankton tend to accumulate at the bottom of the pond (Boyd, 1995; Páez-Osuna et al., 1997; Funge-Smith and Briggs, 1998; Avnimelech and Ritvo, 2003). In fact, only 46.7% of the nitrogen and 7.4% of the phosphorus in the pellets are assimilated by shrimp in semi-intensive farming (Páez-Osuna et al., 1997), leading to the accumulation of huge quantities of organic waste in the pond sediments. Excessive accumulation of organic waste in the sediment during rearing leads to eutrophication of the pond ecosystem, which can encourage the development of diseases in shrimp, resulting in high mortality (Funge-Smith and Briggs, 1998). Indeed, the anaerobic decomposition of the accumulated organic matter leads to the release of hydrogen sulfide (H_2_S) and ammonia (NH_4_^+^), which are toxic for the pond ecosystem. In New Caledonia, at the end of each production cycle, the ponds are usually completely drained to discard the polluted water and then dried in the sun for several months (Boyd, 1995; Yang et al., 2017). The aim of this drying period is to accelerate the aerobic microbial decomposition of the organic waste accumulated in the sediment (Boyd and Pippopinyo, 1994). Thus, this preparation and regeneration of the aquaculture pond between two rearing cycles is therefore a vital step to ensure the success of the next rearing (Della Patrona and Brun, 2009). However, excessive pond drying period may on the contrary limit the microbial decomposition as water stress is reported to narrow the microbial activities (Schimel, 2018). Otherwise, it was reported that for an optimal organic matter degradation, the optimum range of shrimp pond sediment moisture content should be maintained between 10 and 20% and the pH around 7.5–8 (Boyd and Pippopinyo, 1994; Della Patrona and Brun, 2009). As a result, the drying period used by shrimp farmers may not always be effective if the physical and chemical parameters that encourage optimal microbial life and activity are not taken into account. In New Caledonia, ponds can dry out for up to 6 months, which represents a considerable amount of time without economic production for farmers. This can be explained by the seasonal nature of shrimp farming, which means that some farmers only have one production cycle a year (Goarant et al., 2004). However, since few years, New Caledonia shrimp farming face important production decrease, from a peak at 2500 t in 2004 to less than 1,500 t nowadays leading to a negative economic impact (FAO data base “Fisheries and Aquaculture”). It is therefore necessary to find new ways of improving shrimp farming production.

In New Caledonia, salt tolerant plants called halophytes such as glasswort (*Sarcocornia quinqueflora*), austral seablite (*Suaeda australis*) and sea purslane (*Sesuvium portulacastrum*) grow naturally at the vicinity of the shrimp farms (e.g., on the dikes of earthen-ponds) (Colette et al., 2022). In addition, halophytic species rapidly colonize pond sediments when they are abandoned or left empty for several months (Della Patrona, personal communication). Thus, the cultivation of halophytes has recently been explored as a means of improving the quality of pond bottoms by reducing the organic waste accumulated in the sediments at the end of a shrimp rearing cycle (Colette et al., 2022, 2023). The integration of plant cultivation into aquaculture farming systems to limit eutrophication of the aquaculture ecosystem use waste product derived from aquaculture activities to produce plant biomass, and therefore reduce waste concentrations in farming system (e.g., water or sediment) (Miranda et al., 2008; Mariscal-Lagarda et al., 2014; Mariscal-Lagarda and Páez-Osuna, 2014; Yaobin et al., 2019). The nutrient removal of organic matter accumulated in sediments cannot rely solely on plant nutrition. In fact, microbial transformation is crucial for plant nutrition, because in the soil, most nutrients such as N, P and S are bound to organic molecules and are poorly bioavailable to plants (Thies and Grossman, 2006; Jacoby et al., 2017). Microbial communities play a key role in the decomposition of organic matter and the biogeochemical cycling of nutrients in ecosystems (Allison et al., 2010; Luo et al., 2017). Thus, to evaluate the nutrient removal effectiveness of shrimp sediments by halophytes, the microbiota associated with the rhizosphere of plant species must be taken into account (Li et al., 2022). In a previous study, we have demonstrated that the microbiota associated with the rhizosphere of halophytes growing in shrimp sediments varied according to halophyte species (*Sarcocornia quinqueflora, Atriplex jubata,* and *Suaeda australis*) (Colette et al., 2023). In addition, microbial guilds selected by the plants were differently involved in functions linked to the N, C and S biogeochemical cycles in the sediment. However, this previous study was conducted using only one type of shrimp pond sediment. It would therefore be interesting to assess whether the nutrient removal capacity of halophyte species also varies according to the different types of shrimp pond sediment. To do this, we chose to focus on *Suaeda australis*, as this species with a deep root system proved to be effective at eliminating nitrogen (Colette et al., 2022, 2023). This study therefore aimed to assess (i) the impact of *Suaeda australis* and its associated microbiota on both sediment chemistry and microbial communities, (ii) and whether the effect of *Suaeda australi*s and its microbiota on the biochemical parameters of sediments varied according to the shrimp farm sediments. We also aimed to determine whether the initial characteristics (chemical composition and active microbial communities) of the sediments influenced the effectiveness of *Suaeda australis* and its associated rhizosphere microbiota in removing sediment nutrient. To answer these questions, we used four different shrimp farm sediments whose chemical composition we compared in the initial state and either after six months of *Suaeda australis* culture or after six months of drying. The active microbial communities of the sediment and halophyte rhizosphere were also explored by sequencing the cDNA of the V4 region of the 16S RNA gene (Cristescu, 2019; Wood et al., 2020).

## 2. Materials and methods

### 2.1. Greenhouse experiment

The experimental greenhouse is located on a shrimp farm (Aigue Marine) in Boulouparis, New Caledonia, bordering Saint-Vincent Bay. The experiment extended from September (2021) to February (2022) in the same meteorological and watering conditions as in our previous study described in (Colette et al., 2023). Sediments from four different shrimp farms were collected with a medium-sized excavator at the end of the shrimp rearing, on the first days of the drying period of the ponds. In order to keep the names of the shrimp farms anonymous, we will refer to them as A, D, F, and P. The sediments collected were transported to the greenhouse and stored for a few days before being poured into 42 L pots. Two-month-old seedlings of *Suaeda australis* were planted on these sediments. The seedlings were obtained from germinations of seeds from mother plants grown in another experimental greenhouse in New Caledonia (Colette et al., 2023). Then, to avoid drastic change, the young seedlings were transplanted with a part of their initial growth substrate in the 42 L pots filled with sediment. To ensure minimum survival of the halophytes, 3 to 4 young seedlings per pot were planted in the 42 L pots. For each sediment from each farm (A, D, F and P), 9 pots were used for the cultivation of *S. australis*.

The old plants were grown for 6 months in the greenhouse and irrigated daily using an automatic sprinkler system. We chose a cultivation period of 6 months for our experimentation as it is the duration of pond sediment drying practiced by shrimp farmers in New Caledonia (Goarant et al., 2004). Twice a week, *S. australis* were watered with lagoon seawater used for the shrimp farm activities. For each sediment from each farm (A, D, F and P), 3 pots of 42 L were also used for a dry treatment condition. This consisted of pots containing only pond sediment, placed outside the greenhouse without watering for the 6 months of the experiment to mimic the effect of drying out the pond (dry conditions). During the drying period used by shrimp farmers, the bottom of the emptied ponds is dried in the sun (Boyd, 1995; Della Patrona and Brun, 2009). Thus, the pots were exposed to the same weather conditions as the sediments of the emptied ponds and subjected to the natural drying period.

### 2.2. Samples collection

#### 2.2.1. Sampling for chemistry analysis

Sampling was carried out at the beginning (D0) and at the end of the 6 months experiment. For each farm (A, D, F and P), the sediment was sampled in the 42 L pots on D0 (before the culture of *S. australis*) and at the end of the experiment in the dry condition (Dry) and in sediment with *S. australis* cultivation. For the chemical analyses, sediment of each farm (A, D, F and P), were sampled in triplicates in different 42 L pots from each modality (D0, dry, *S. australis*). To ensure sampling homogeneity, each sediment samples consisted of a pool of 6 samples from different pots, homogenized in a clean bucket and then stored in aluminum trays and 50 mL tubes. The collected sediment in aluminum tray was then oven-dried at 35°C for several days for analysis of the pH, total and available forms of phosphorus, total sulfur and organic carbon. The other part of the sediment collected in the 50 mL tubes were stored at −20°C for the analysis of available nitrogen forms (NO_3_^−^ and NH_4_^+^).

#### 2.2.2. Sampling for microbial communities analysis in the sediment

Several publications have used cDNA metabarcoding and proved that RNA is a useful tool to identify living organisms and to perform biological survey and monitoring (Laroche et al., 2018; Amarasiri et al., 2021; Miyata et al., 2021; Veilleux et al., 2021). Indeed, the high turnover of RNA molecules in the environment (from days to weeks) compared to DNA (from months to years) reflect better the metabolically active lineages at the sampling time. Thus, in our research, we opted to extract RNA to investigate the active microbiota in the rhizosphere and sediment, minimizing the chances of detecting microorganisms that may be inactive or dead in the samples. In order to explore the active microbial diversity in the sediment, the top 2–3 cm of sediment from the 42 L pots were also collected aseptically using RNA/DNA free gloves and spatula. For each farm (A, D, F, and P), the sampling was carried out at the same time as sampling for chemical analyses at D0 and in the Dry and *S. australis* conditions. We collected sample in triplicate and one replicate consist of 3 to 4 sediment samples from the same pot. Then, sampled sediment was transferred into RNA/DNA free 15 mL tubes. The collecting sediment were stored at 4°C during transport to the laboratory and then frozen at - 80°C until further processing.

### 2.3. Sediment chemistry analysis

The Laboratory of Analytics Means (LAMA/ISO 9001, Noumea, New Caledonia) performed the analyses of cation exchange capacity (CEC), calcium carbonate (CaCO_3_), pH, organic carbon (C.org), nitrate (NO_3_^−^), ammonium (NH_4_^+^), total and available forms of phosphorus and total sulfur. The CEC was measured by the cobaltihexamine chloride methods (ISO 23470) and the CaCO_3_ with a Bernard calcimeter. Sediment pH was measured with pH electrod SCHOTT Blue Line in soil/distilled water ratio of 1:2.5. The nitrate (NO_3_^−^) and ammoniums (NH_4_^+^) were extracted from the sediment with KCl solution at 1 N. The nitrate and ammonium concentrations were evaluated by colorimetric method based on the Griess reaction (ISO 14256-2:2005) and Nessler method (ISO 14256-2:2005) respectively. Total organic carbon was determined using the Walkley and Black method (Pétard, 1993). Total phosphorus was measured by Murphey and Riley method (Murphy and Riley, 1962) whereas available phosphorus forms by Olsen method (Olsen, 1954). Total sulfur was determined after alkaline fusion by ICP-OES. A non-parametric test of Kruskal-Wallis followed by a Dunn’s test were performed with R software to show statistically significant differences of sediment chemistry between the experimental conditions.

### 2.4. Microbial communities in the sediments

#### 2.4.1. RNA extractions, retro-transcription, and sequencing

For each sediment sample, RNA was extracted using RNA PowerSoil Total RNA Isolation Kit (MoBio Laboratories, Inc.) and then reverse-transcripted into complementary DNA (cDNA) as described in our previous study (Colette et al., 2023) using Second Strand cDNA Synthesis Kit (Invitrogen). All cDNAs were sent to MrDNA (Shallowater, Texas, United States) where PCR using the 515f-806R primers couple (Caporaso et al., 2011), barcode indexing and sequencing of the V4 hypervariable region of the reverse-transcripted procaryotic 16S rRNA molecule were carried out. The sequencing was done with an average of 20 k raw reads per sample.

#### 2.4.2. Downstream analysis

The amplicon analysis was performed with DADA2 version 1.6 package^1^ on R software as described in Colette et al. (2023). The chimeras were removed using the consensus method, and the taxonomy was assigned using the Silva 138 SSU Ref NR99 database (Quast et al., 2012). Sequences with no affiliation or affiliated to the Eukaryota, Mitochondria or Chloroplasts were removed from the ASV table, prior to further analysis.

The alpha diversity of each sediment sample was calculated on R software with the *microeco* (v0.20.0) package (Liu et al., 2021). Then, data were normalized with the Counts Per Million (CPM) as described in Callac et al. (2022, 2023). The beta diversity was investigated by a PCoA (principal coordinate analysis) plot using the Bray distance with the *microeco* package on R software. Then, a permutation test (PERMANOVA) was performed to highlight Bray distances significantly different between farms (A, D, F and P) and modalities (D0, *S. australis* and Dry). Venn diagrams were then made to exhibit both shared and specific ASVs between the farm sediments for each of the three different modalities (D0, dry and *S. australis*). The Venn diagrams were built using the open-source component for the web environment Jvenn^2^. For each modality, stacked bar charts of the relative abundance of microbial communities were made to display the composition of the sediment microbial communities according to the farms sampled. Stacked bar plot were performed on R software with *microeco* and *ggplot* (v3.4.2) packages. For the *S. australis* modality, we performed a Functional Annotation of the Prokaryotic Taxa to predict the putative functions of the specific sedimentary microbial communities on each farm. The functional annotation was based on FAPROTAX v1.2.4 database and was done on R software with *microeco* package.

## 3. Results

### 3.1. Comparisons of sediment chemical parameters between shrimp farms

#### 3.1.1. At the beginning of the experiment

At the beginning of the experiment (D0), the chemical parameters of the sediments varied from farm to farm (Table 1). With the exception of sediment from farm D, which had a pH of 7.1, the pH of farms A, F and P was around 8 (Table 1). Sediment CEC values ranged from 23.7 to 30.4, with the highest value observed on farm D.

**Table 1:** Average of sediment chemical parameters across four shrimp farms named A, D, F, and P, at the beginning of the experiment (D0) and after 150 days of *S. australis* cultivation or in dry sediment.

	Farm	CEC (meq 100 g^−1^)	CaCO_3_ (%)	pH	[NO_3_^−^] mg.kg^−1^		[NH_4_^+^] mg.kg^−1^		C.org mg.g^−1^		C/N	P.ass mg.kg^−1^		P.tot mg.kg^−1^		Sulfur mg.kg^−1^	
		
	A	23.7	1.5	8^ab^	14.7^ab^		5^ab^		8.6^a^		10^a^	83.3^ab^		661.3^a^		1841.6^a^	
D0	D	30.4	2.3	7.1^a^	11.5^a^	Variation compare to D0 (%)	10.7^a^	Variation compare to D0 (%)	8.4^a^	Variation compare to D0 (%)	8^ab^	91^ac^	Variation compare to D0 (%)	751.7^ab^	Variation compare to D0 (%)	2040.2^ab^	Variation compare to D0 (%)
	F	27.6	27.2	8.4^c^	16.3^ab^		1.7^b^		9.3^a^		12^a^	55^b^		730.3^ab^		2429.4^bc^	
	P	29.0	34.67	8.3^bc^	**41.6**^b^		3.9^ab^		8.8^a^		7^b^	97.3^c^		817.7^b^		2625.2^c^	
*S. australis*	A			8.2^ab^	1.3^ab^	−91%	2.9^a^	−42%	9.9^a^	+15%	10^ab^	39^ab^	−53%	617^a^	−7%	1419.6^a^	−23%
	D			7.8^a^	**27.1**^**a**^	**+136%**	2.6^a^	−76%	12.3^a^	+46%	12^a^	44.7^a^	−51%	659.3^ab^	−12%	2778.4^a^	+36%
	F			9^ab^	0.2^b^	−99%	2.2^a^	+29%	11.6^a^	+25%	13^a^	18.7^b^	−66%	626a^b^	−14%	913.1^a^	−62%
	P			8.5^ab^	20.2^a^	−51%	2.1^a^	−46%	10.2^a^	+16%	9^b^	37^ab^	−62%	693^b^	−15%	3040.8^a^	+16%
	A			9^ab^	12.2^ab^	−17%	2.1^a^	−58%	8.6^a^	0%	10^a^	30.7^ab^	−63%	620^a^	−6%	429.9^a^	−77%
Dry	D			7.3^a^	**24.2**^**a**^	**+110%**	**3.4**^**b**^	**−68%**	11.9^b^	+42%	11^a^	41^a^	−55%	683.7^ab^	−9%	2953.5^b^	+45%
	F			9.4^c^	3^c^	−82%	2.6^ab^	+53%	10.9^b^	+17%	11^a^	22.3^b^	−59%	830^c^	+14%	1093.1^b^	−55%
	P			9.4^bc^	5.9^bc^	−86%	2.6^ab^	−33%	9.5^ab^	+8%	10^a^	30^ab^	−69%	812.3^bc^	−1%	1026.6^ab^	−61%

The gray boxes represent percentage of variation of each parameter compared to D0. a, b, and c: show statistical significance between all D0, all *S. australis* and all dry sediments, determined by a non-parametric test of Kruskal–Wallis followed by a Dunn’s test.

Farms A and D both had a lower percentage of calcium carbonate (CaCO_3_) in their sediments (1.5 to 2.3%) than farms F and P (27.2 to 34.67%). The C/N ratio of farm sediments ranged from 7 to 12 (Table 1). Sediment organic carbon concentration did not vary significantly between farms, unlike NO_3_^−^, NH_4_^+^, phosphorus and sulfur concentrations. Sediments from farm P had significantly higher concentrations of sulfur, total and available phosphorus, and NO_3_^−^ (Table 1). Sediments from farms A and D had significantly lower sulfur concentrations than those from farms F and P.

#### 3.1.2. Under *Suaeda australis* cultivation

After 150 days of *S. australis* cultivation, sediment pH was still significantly lower in farm D than in the other farms (Table 1). For farms A, F and P, *S. australis* cultivation reduced the NO_3_^−^ concentrations by 50 to 99% compared with D0. For farm D only, *S. australis* cultivation increased sediment NO_3_^−^ concentration by 136% (Table 1). For all farms, *S. australis* cultivation increased the concentration of organic carbon in sediment compared to D0 and decreased the concentrations of phosphorus (total and available forms). Farm D showed the greatest increase in organic carbon (+ 46%) compared with the others farms (+ 15 to 25%). The greatest reduction in phosphorus under *S. australis* cultivation was found in the sediments of the farms F and P (Table 1). In terms of sulfur concentration, *S. australis* cultivation increased concentrations on farms D (+36%) and P (+16%) whereas it decreased on farms A (−23%) and F (−62%).

#### 3.1.3. In dry sediment

Sediment drying resulted in a pH increase of one unit compared with D0 in all farms except farm D (Table 1). Sediment pH values were around 9 on farms A, F, and P, and 7.3 on farm D (Table 1). NO_3_^−^ concentration was reduced by 80% in farms F and P and by 17% in farm A. Conversely, NO_3_^−^ and total sulfur concentration in dried sediments from farm D increased by 110 and 45%, respectively, compared to D0 (Table 1). The dried sediments from farm D showed higher NO_3_^−^ and total sulfur concentrations than those from the other farms, which were not detected at D0 (Table 1). For all farms, sediment drying reduced the concentration of available forms of phosphorus compared to D0, within a range quite similar to that of the *S. australis* cultivation (Table 1). Sediment organic carbon increased in dry sediments from farms D, F, and P compared to D0, but the increase was smaller than under *S. australis* cultivation.

### 3.2. Comparisons of sediment microbial communities

#### 3.2.1. Alpha diversity index

On D0, farms F and P had higher values of richness indices (Chao1 and observed ASV) and evenness indices of Shannon compared to the farms A and D (Table 2). After 150 days of *S. australis* cultivation, the average of all alpha diversity indices decreased in farms F and P compared with D0, while they increased in farm A and F (Table 2). The highest values of alpha diversity indices (Chao1, Observed ASV, Shannon and Simpson) with *S. australis* cultivation were found in farm A sediment. For all farms, average of richness indices decreased in the dry sediment compared with D0. In addition, for all farms, the richness indices values in dry sediments were lower than in sediment with *S. australis* cultivation (Table 2). In the dry sediments, the highest of Chao1 and Observed ASV values were found in farm P.

**Table 2:** Comparison of average alpha diversity indices of richness (Chao1, observed ASV) and evenness (Shannon and Simpson) between the different shrimp farms sediment at the beginning of the experiment (D0) and after 150 days of *S. australis* cultivation or in dry sediment.

	**Farm**	**Chao1**	**Observed ASV**	**Shannon**	**Simpson**
D0	A	3772.8	2858.3	5.7	0.98
	D	3427.3	2475.5	5	0.95
	F	5081.1	3,988	6.6	0.99
	P	5357.8	3,945	6.4	0.99
*S. australis*	A	5221.8	3873.3	6.3	0.99
	D	3685.9	2,902	5.8	0.98
	F	3793.8	2850.5	4.5	0.83
	P	4682.9	3,488	6.1	0.98
Dry	A	2230.5	1757.5	5.7	0.99
	D	1971	1624.5	5.3	0.98
	F	2009.6	1557.5	5.3	0.97
	P	2730.5	2247.5	5.4	0.98

#### 3.2.2. Microbial samples ordination

**Figure 1:**
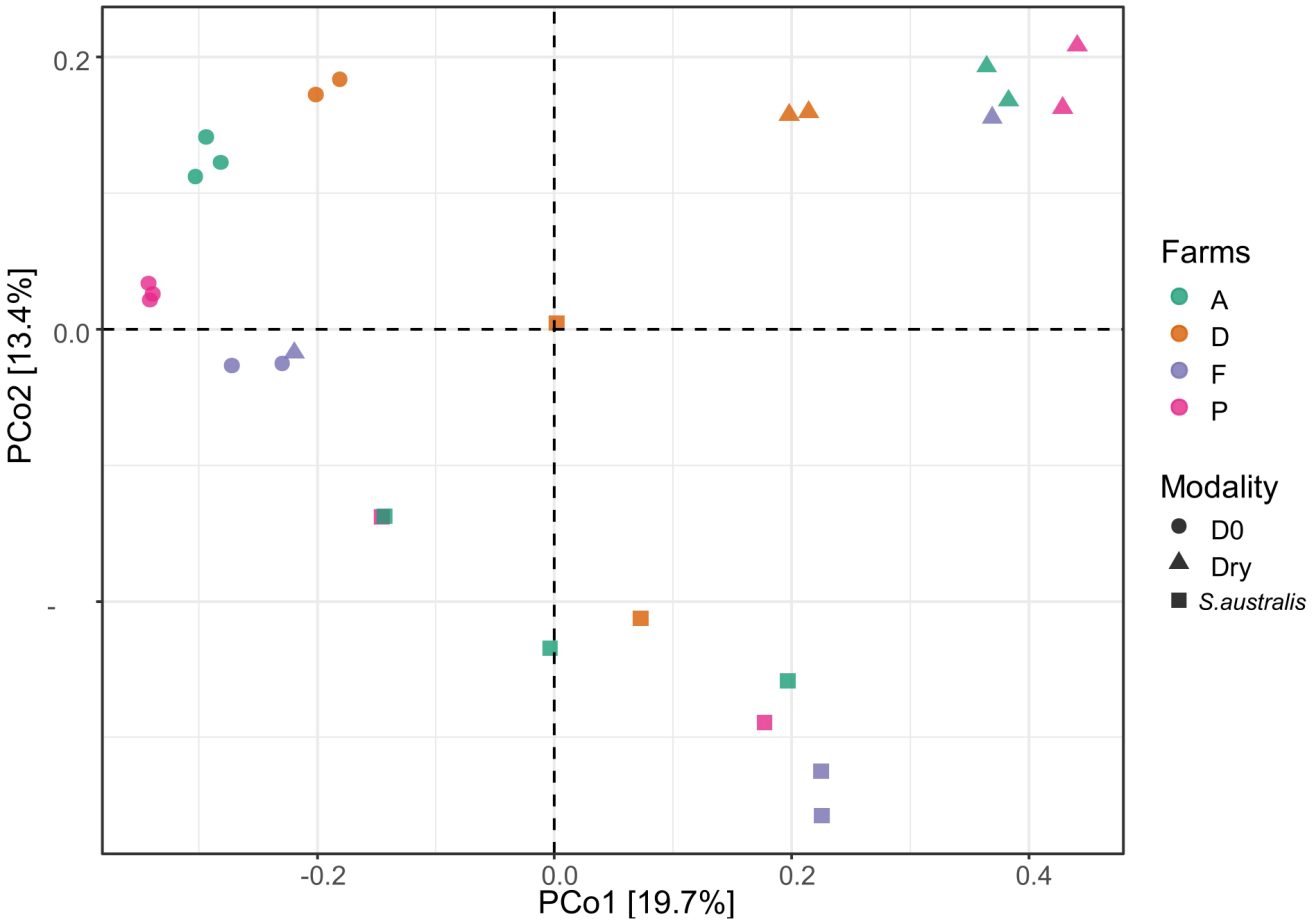
Principle coordinate analysis (PCoA) plot, of the microbial diversity in the sediments from the farms A, D, F, and P following the different modalities (D0, dry, *S. australis*), made using the Bray-Curtis distance.

Our dataset was composed of 20,467 ASVs. The PCoA diagram displayed that the sediment microbiota differed between D0, dry and *S. australis*, as the points representing these different modalities are clearly separated in the graph (Figure 1). In addition, a differential test of distances among groups performed with a PERMANOVA evidenced that those three modalities were statistically different with a *value of p* at 0.001 (Table 3).

**Table 3:** Results of the permutation test (PERMANOVA) based on Bray–Curtis measure for pairwise comparison of farms (A, D, F, and P) and modalities (D0, *S. australis* and Dry).

	p.value	Significance
Farms		
Farm D *vs.* Farm P	0.006	*
Farm D *vs.* Farm F	0.007	*
Farm D *vs.* Farm A	0.027	*
Farm P *vs.* Farm F	0.361	
Farm P *vs.* Farm A	0.055	
Farm F *vs.* Farm A	0.119	
Modalities		
*S. australis vs.* Dry	0.001	***
*S. australis vs.* D0	0.001	***
Dry *vs.* D0	0.001	***

Number of permutations used: 999. *p < 0.05 and ***p < 0.001.

All modalities confounded, the PERMANOVA also evidenced that farm D sediment microbiota was significantly different (value of *p* < 0.05) from farms A, F, and P (Table 3). This can be evidenced in the plot for the modalities D0 where samples from farm D were distant from farms F and P (Figure 1). Furthermore, when considering the dry modality in the PCoA diagram, sediment samples from farm D were isolated from other farms.

For the *S. australis* modality, sediment samples were randomly distributed across the graph (Figure 1). For this modality, there was more distance between replicates of sediment samples, highlighting a greater variability of microbiota within sediments from the same farm. For farm F only, replicates of sediment microbiota samples with the *S. australis* modality were close to each other (Figure 1).

### 3.3. Microbial communities inhabiting – D0 and -dry sediments

**Figure 2:**
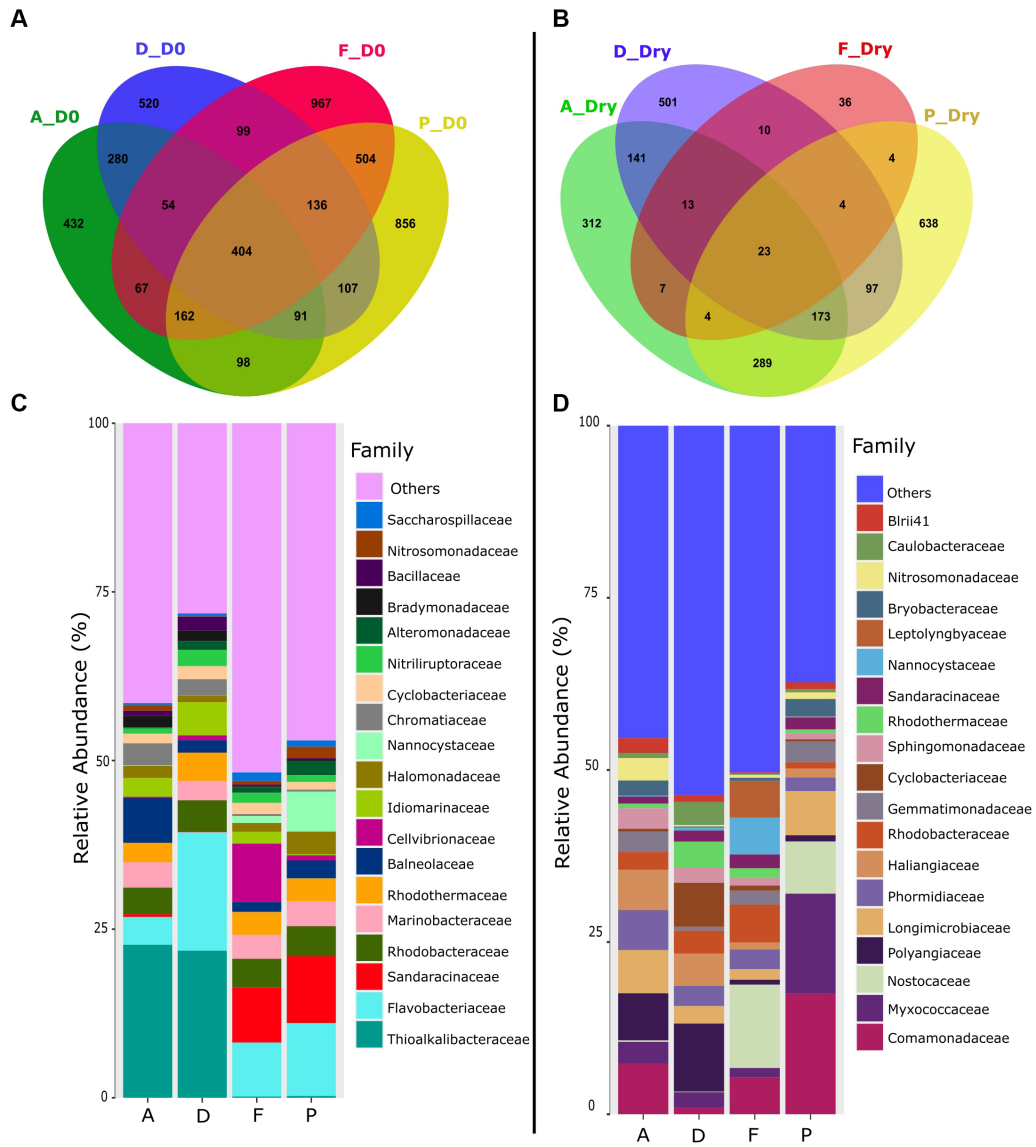
Venn diagrams of shared and specific ASVs in the sediment of farms A, D, F and P, in **(A)** the sediment at the beginning of the experiment (D0), **(B)** in the dry sediment. The relative abundance of the specific ASVs at the bacterial family level found in each farms sediments was represented in **(C)** at the beginning of the experiment and in **(D)** dry sediment.

Venn diagram showed that at the beginning of the experiment (D0), microbiota of the four farms shared 404 ASVs (Figure 2A). At D0, the farms F and P shared 504 ASVs and fewer ASVs with farm A (67 and 98 ASVs respectively), while farms A and D together shared 280 ASVs.

On D0*, Thioalkalibacteraceae* was the main bacterial family found in the sediments of the farms A and D with a relative abundance of about 22% whereas this family accounted for less than 0.27% in the farms F and P (Figure 2C and Supplementary Table S1). The *Chromatiaceae* family was also mainly found in the farms A and D sediments with relative abundance around 3% whereas it was less than 0.3% in the other farms. Furthermore, on D0, microbiota of the farms F and P was composed of *Sandaracinaceae* (between 8 and 9%) whereas this family was less than 0.5% in the farms A and D.

For the four farms on D0, the active microbiota was composed of members of the *Flavobacteriaceae*, *Marinobacteraecae, Rhodobactacteraceae,* and *Rhodothermaceae* families. The microbiota of the farm F was also composed of *Cellvibrionaceae* (8.65%) which was represented less than 1% of the microbial abundance in the sediments of the other farms (Figure 2C and Supplementary Table S1). The microbiota of the farm P was also composed by *Nannocystaceae* (6%), which encompassed less than 1% in the other farms.

In dry conditions, however, the Venn diagram showed that only 23 ASVs were shared between the four farms (Figure 2B). In the dry sediment, among the 19 main families identified, only 5 were present at D0 (*Nannocystaceae, Sandaracinaceae, Rhodotermaceae, Cyclobacteraceae, Rhodobacteraceae,* Figure 2D). *Polyangiaceae* and *Haliangiaceae* families were present in the farm A (6.8%) and D (9.9%) but accounted for less than 1% in the farms F and P (Figure 2D and Supplementary Table S2). The *Nostocaceae* family was present in sediments of the farms F and P; but was absent in farms A and D. *Comamonadaceae* family was found in sediments of the farms A (7%), farms F (5%) and farm P (17%) but represent only 1% in farm D. The *Nitrosomonadaceae* family was found in farm A with a relative abundance of 3% but was less than 1% in other farms. The dry sediment of the farm P was enriched in *Myxococcaceae* (14.5%) compared to others farms (less than 3%). Farm F was composed of *Leptolyngyaceae* (5%) and *Nannocystaceae* (5%) whereas the relative abundance of those families were less than 0.4% in the dry sediments (Figure 2D and Supplementary Table S2).

### 3.4. Specific microbiotas in the sediment with *Suaeda australis* and the associated putative microbial functions

#### 3.4.1. Specific microbiota

**Figure 3:**
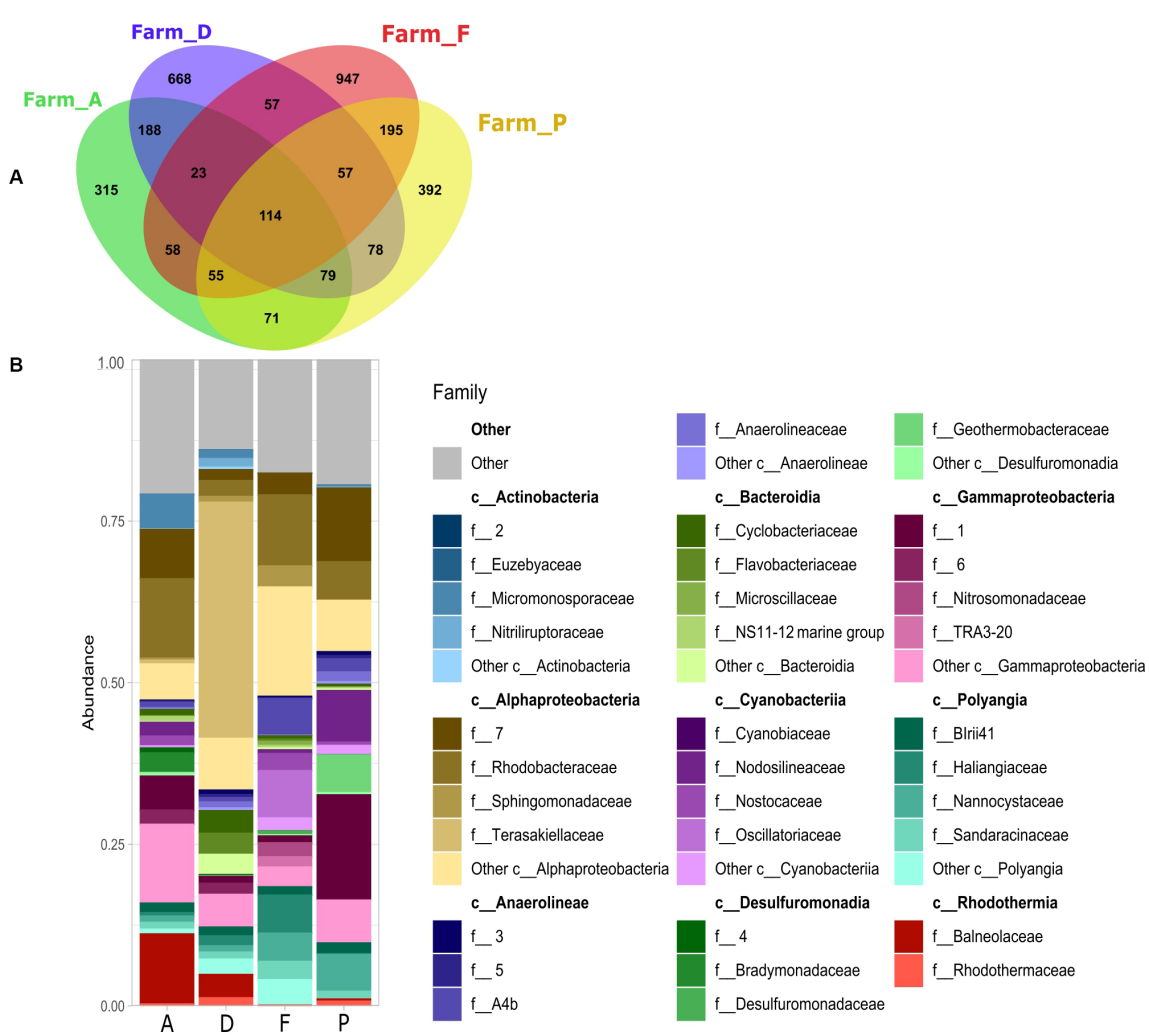
**(A)** Venn diagram of shared and specific ASVs in the sediments with *S. australis* from the farms A, D, F, and P. **(B)** Stacked bar plot represent relative abundance of the 10 main bacterial classes and the 5 main families per class, of the specific ASVs found in each farm.

At the family level, the specific microbiota was varying between the farms (A, D, F and P) (Figures 3A,B). In the farm A, specific microbiota was composed of *Balneolaceae* (10%), *Micromonosporaceae* (5%) and *Bradymonadaceae* (3%). Specific microbiota of farm D was mainly composed of *Terasakiellaceae* (36%) family which was absent in the other farms (Figure 3B and Supplementary Table S3). In the farm F, the specific microbiota contained members *Oscillatoriaceae* family (7%) which was totally absent in the other sediments, and *Sphingomonadaceae* (3*%*) which was less than 1% t in the other farms. The specific microbiota of the farm F was also composed by lineages related to the *Haliangiaceae* (6%) and *A4b* (Fermentative organism found inside of anammox granules) (5%) which accounted for less than 2% in the specific microbiota of the others farms. The specific microbiota of the farm P was composed of *Nodosilineaceae* (8%) in greater proportion than in other farms, and of *Nannocystaceae* (5%) which was also present in the farm F (4%) and in lesser proportion (< 1.5%) in the farms A and D. The specific microbiota of the farm P also included the *Geothermobacteraceae* family (6%), which was absent from the other farms.

*Rhodobacteraceae* was found in the specific microbiota of all farms, with relative abundance of 3% in the farm D, of approximately 12% in the farms A and F and about 6% in the farm P (Figure 3B and Supplementary Table S3).

#### 3.4.2. Putative functions of *Suaeda australis* specific microbiotas

**Figure 4:**
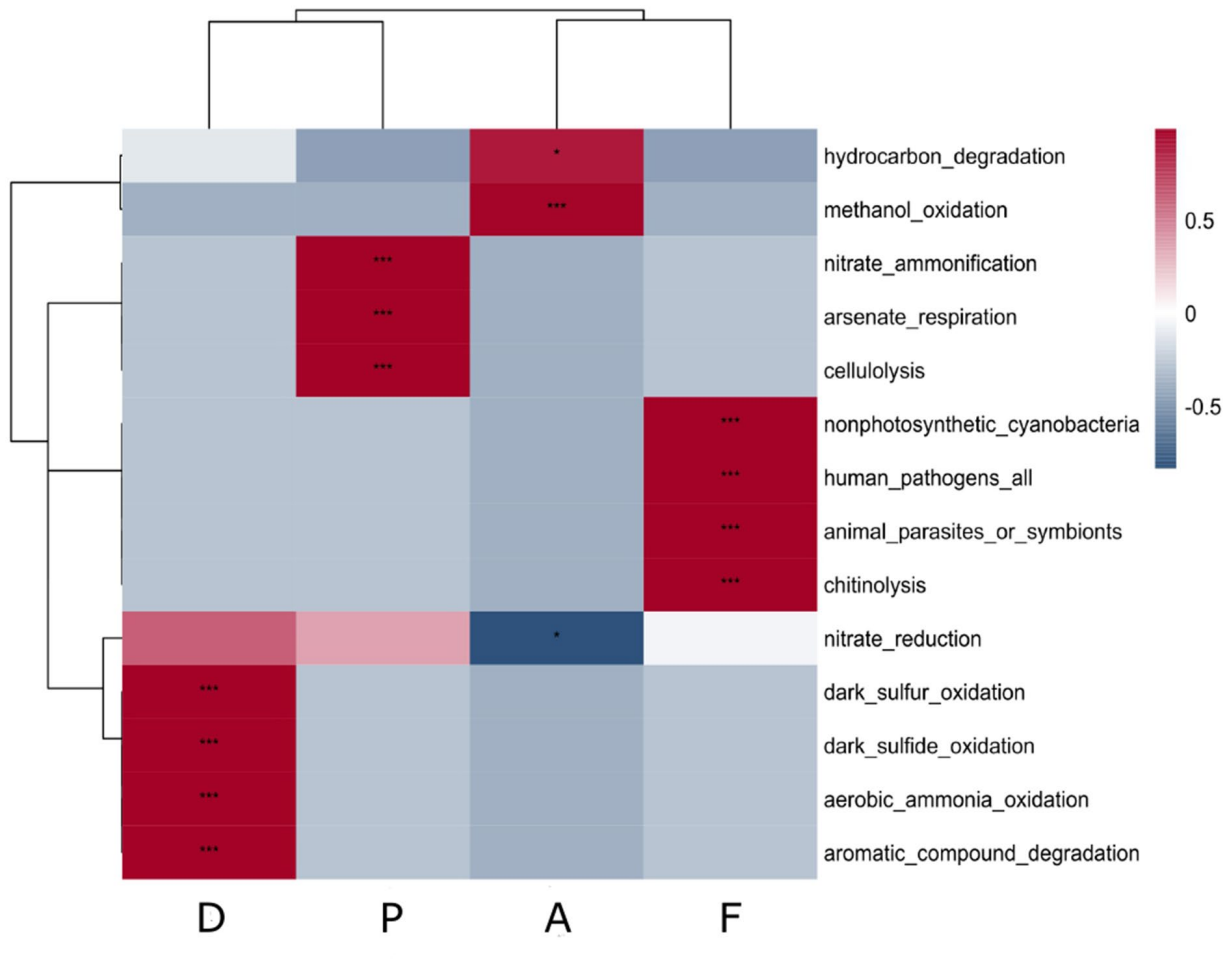
Correlation heatmap of putative ecological function associated with the specific microbiota of *S. australis* according to the farm sediment. Heatmap color gradient is linked to Pearson correlation coefficient intensity with in red the positive correlation and in blue the negative correlation. Significant correlations between ecological function and farms are indicated by an asterisk (*).

In the farm D, the putative functions of the specific microbiota of the sediment with *S. australis* were significantly and positively correlated with the sulfur cycle (sulfide and sulfur oxidation), ammonia oxidation and aromatics degradation (Figure 4). For farm A sediment microbiota, correlations were significantly positive with hydrocarbon degradation and methanol oxidation.

In the farm P, the putative functions were significantly positively correlated with nitrate ammonification, arsenate respiration and cellulolysis. Other lysis functions, such as chitinolysis, were also found as putative functions in farm F (Figure 4). However, in the sediment microbiota from Farm F, significant positive correlations were also found.

#### 3.4.3. Evolution of principal bacterial classes in the rhizosphere during the experiment

**Figure 5:**
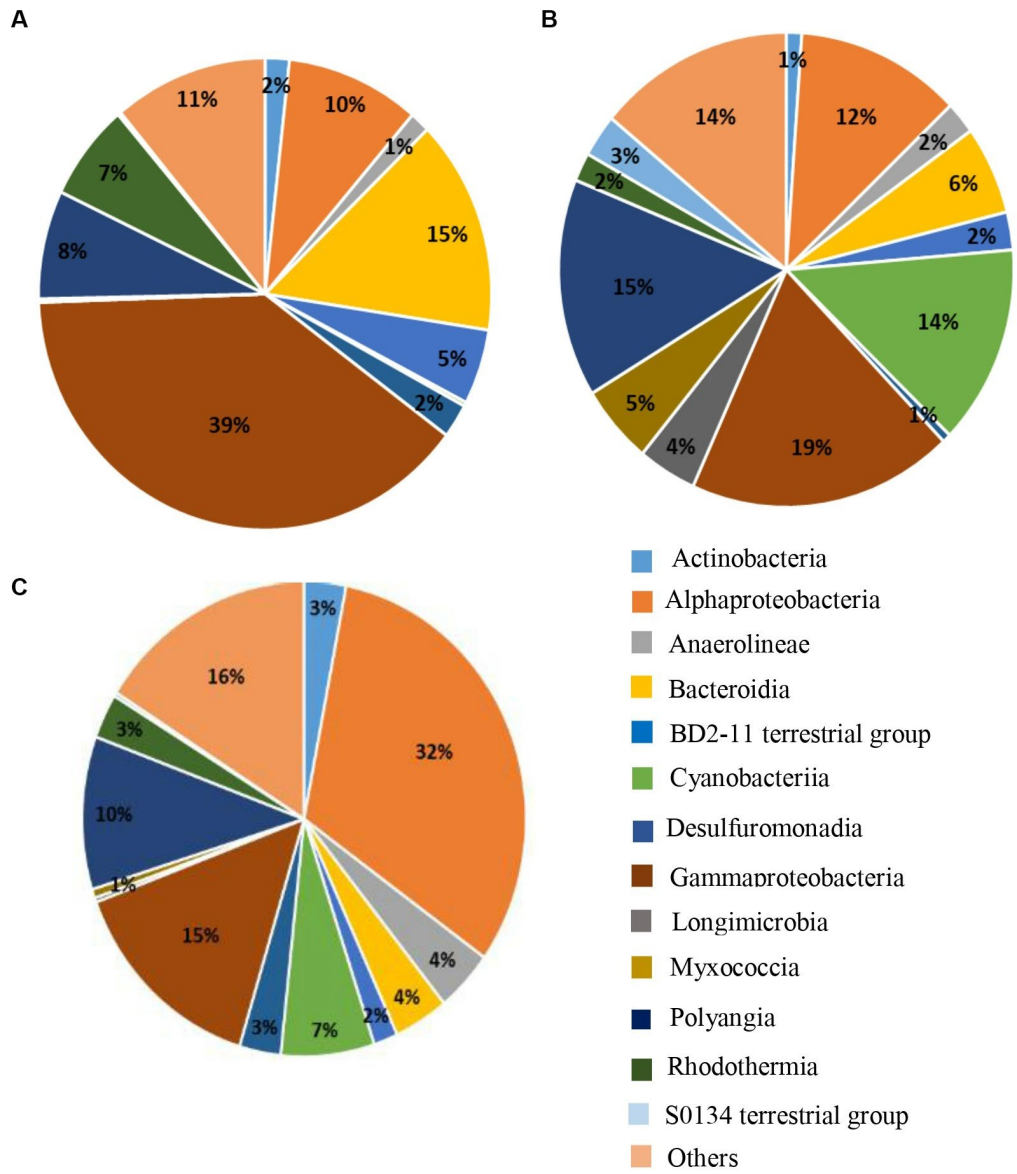
Pie charts of the 15 main bacterial classes representing at least 84% of the relative abundance found in the microbiota of the sediment at **(A)** D0, **(B)** in the dry sediment, and **(C)** in the rhizosphere of *S. australis*, all farms combined.

We could observe that at D0 and for all the farms, the *Gammaproteobacteria* was the main class that dominated the sediment microbiota (39%) followed by the *Bacteroidia* (15%) (Figure 5A). However, in the dry conditions (Figure 5B), the abundance of those two bacterial classes decreased by a factor two. In the dry conditions the relative abundance of the *Polyangia* increased compared to D0 (8% compared to 15%) and the *Cyanobacteria* rise in great proportion (14%) (Figure 5B). The *Myxococcia* and *Anaerolineae* found in the dry sediment were absent at D0. In the rhizosphere of *S. australis*, the relative abundance of *Alphaproteobacteria* had significantly increased compared to the others conditions and constituted the main bacterial class of the rhizosphere microbiota (32%) (Figure 5C). *Cyanobacteria, Polyangia, Bacteroidia* and *Myxococcia* were also present in the *S. australis* rhizosphere but in a lesser proportion than in the dry sediment.

## 4. Discussion

The aim of this study was to evaluate the impact of *Suaeda australis* and its associated active microbiota on sediment chemistry and microbial communities depending on the sediment composition of four shrimp farms (A, D, F, and P). For that we have compared the change of sediment chemistry and active microbial communities from each farm at D0 and in the presence or not of halophyte cultivation. In the absence of halophytes, the sediment was left drying outside the greenhouse along with the experiment to mimic the pond drying periods practiced by shrimp farmers. By comparing these two conditions (with and without halophytes), we were able to highlight the changes caused by plants and their rhizosphere microbiota, and how these vary between the sediments of shrimp farms.

### 4.1. Characterization and comparison of shrimp farms sediments at D0

At the beginning of the experiment (D0), chemical parameters of sediment varied between the farms (Table 1). These variations can be easily explained primarily by differences in aquaculture practices on the farms during the rearing cycles as shrimp density, feeding rate, fertilization (N, P) and liming strategy (CaCO_3_). These parameters can strongly influence the concentration of nutrients in the pond sediment, such as nitrogen, carbon, phosphorus and sulfur (Boyd, 1995; Avnimelech and Ritvo, 2003). The C:N ratio found in sediments at the beginning of the experiment was varying between 7 and 12, and these values were within the range of those reported in others studies of aquaculture pond soils (Munsiri et al., 1995; Smith, 1996). These C:N ratio values were recorded in the case of rapid biological mineralization of the soil, which means that overall, in our study, the organic matter (feed pellets, feces) in the sediments at D0 must have been easily decomposed by the microorganisms (Brust, 2019). In our study, the farms F and P were chemically distinct from farms A and D with higher concentration of CaCO_3_ in their sediments at the end of the rearing (Table 1). Farms F and P also showed the highest values of alpha diversity richness indices (Chao1, observed ASV) on D0, compared to farms A and D (Table 2). It would be therefore interesting to study the effect of liming on the microbial communities in shrimp pond sediments to explore if this is a factor that could have directly influenced the differences in alpha diversity indices between the studied farms. In fact, in terrestrial soil, previous studies have reported that a proper application of CaCO_3_ have enhanced the microbial alpha diversity indices (Shannon, Simpson) in acid paddy-field (Guo et al., 2019) and microbial richness indices (Observed ASV, ACE) in sugarcane field (Pang et al., 2019). In shrimp ponds, the main source of sediment calcium carbonate comes from liming which is a common practice to improve the pH and alkalinity of aquaculture ponds (Boyd, 1995; Zhou et al., 2019). It was reported that sediment pH has a pronounced effect on microbial organic matter decomposition. Indeed, Li et al. (2015), reported that below a pH value of 7.5, liming of shrimp pond sediments is necessary to increase the microbial respiration rate and therefore organic decomposition during the drying period. Thus, from the start of the experiment, the pH values found in the sediments of farm D may not be conducive to microbial decomposition of organic matter, unlike the other farms (Table 1).

At the beginning of the experiment, the four shrimp farms sediment were all characterized by the occurrence of *Marinobacteraecae, Rhodobactacteraceae,* and *Rhodothermaceae. Flavobacteriaceae* also occurred in all the shrimp farm sediment but with a higher relative abundance in farms D and F than in farms P and A. *Flavobacteria* family was commonly found in freshwater, marine and terrestrial environments (Rosenberg, 2014). They are chemoorganotrophic bacteria found associated with algae cells, fish or also organic detritus. The *Rhodobacteraceae*, *Flavobacteraceae, Marinobacteraceae* families were previously found as dominant taxa families in the shrimp larval rearing water of New Caledonia (Callac et al., 2022, 2023) suggesting that these taxa were usually detected in shrimp rearing. In addition, *Rhodobacteraceae* and *Flavobacteraceae* were also found, respectively, as microbial biomarker of shrimp gut and pond sediment samples (Zhou et al., 2021) suggesting that those taxa were commonly associated with shrimp farming ecosystems.

Farms A and D were characterized by the dominance of *Thioalkalibacteraceae* in their sediment whereas this family was absent in farms F and P (Figure 2A). This family is known to use thiosulfate, elementary sulfur and sulfide as electrons donors (Boden, 2017). The sediment microbiota of those two farms were also composed of *Chromatiaceae* family which was also reported to be involved in sulfide and elemental sulfur oxidation (Imhoff, 2014). The sediments from these two farms had the lowest total sulfur concentration compared with farms F and P, suggesting a potentially important role for *Thioalkalibacteraceae* in the elimination of sulfur from these sediments.

Farms F and P sediment were mainly characterized by the occurrence of *Sandaracinaceae* family from the *Myxobacteria* phylum. Members of this bacterial phylum are reported to have the ability to lyse living cells of other microorganisms by lytic enzyme and to be cellulose-decomposers (Mohr et al., 2012; Mohr, 2018). The *Nannocystaceae* was another bacterial family belonging to *Myxobacteria* phylum and was particularly found in farm P sediment but was absent in farms A and D. This family was reported to be capable of degrading complex macromolecules and lysing microorganisms (Garcia and Müller, 2014). Because of their lytic capacity, *Myxobacteria* are considered as an important micro-predators playing a key role in structuring and regulating soil microbial communities (Shimkets et al., 2006; Phillips et al., 2022). The presence of this taxon was strongly influenced by bacterial communities and was previously reported to be significantly positively correlated to alpha diversity indices of Chao1 and Observed OTU (Dai et al., 2021) and of ACE, Shannon and Simpson (Wang et al., 2020). Thus, in our study the higher alpha diversity indices (observed ASV, Chao1 and Shannon) may have promoted the occurrence of the *Myxobacteria* in farms P and F by given them a broader choice of potential prey microorganisms. The microbiota of the farm F had the particularity to be composed of *Cellvibrionaceae* family whereas this family was nearly absent from the others farms. A widespread trait among strains of this family was reported to have the ability to use complex polysaccharides as substrates (Spring et al., 2015). This family was identified as highly productive of carbohydrate-active enzymes that can be involved in lignocellulose degradation (Leadbeater et al., 2021). Thus, a characteristic of the microbiota of the farms F and P at the beginning of the experiment was the occurrence of bacteria with lytic and complex organic matter degradation capabilities.

### 4.2. Changes in sedimentary microbiota of the different shrimp farms under dry conditions

After the 150 days of experimentation in pots, the sediment microbiota has evolved differently from D0 regarding the dry or *S. australis* modalities (Figure 1 and Table 3). Drying had significantly decreased the alpha diversity indices in the sediment of each farm compared to D0 (Table 2). Regarding the drying effect on the microbial diversity, it had been widely reported that prolonged drought has a significant impact on the abundance, structure and activities of the soil microbiome (Bogati and Walczak, 2022). In the soil, water is a solvent and a transport medium of microbial substrates so it has a direct influence on the ability of bacteria to acquire soil substrates. In addition, soil water can also directly influences the physiological state of bacteria (Chen et al., 2007; Schimel, 2018). *Myxobacteria* was a phylum highly represented in the dry sediments with 5 families: *Nannocystaceae, Myxococcaceae, Haliangicaeae, Polyangiaceae, Sandaracinaeceae,* out of the 19 main families observed (Figure 2D). In dry sediments, the *Myxobacteria* phylum represented by *Myxococcia* and *Polyangia* classes, had higher relative abundance than in D0 (Figure 5B). Their significant occurrence in dry sediments can be explained by the fact that *Myxobacteria* are spore-forming micro-organisms that can become dormant under stressful environmental conditions like dryness (Dawid, 2000; Mohr, 2018). The families related to *Myxobacteria* was varying within farms sediment with *Haliangiaceae* and *Polyangiaceae* specifically related to farms A and D, *Nannocystaceae* to farm F and *Myxococcaceae* to farm P (Figure 2D). Several *Cyanobacteria* families were also found in the microbiota in the dry sediment of each farms. However, the relative abundance of the different families also varied between the farms. Thus, *Nostocaceae* were present in farms F and P but in lesser extent in farms A and D, the *Leptolyngbyaceae* were only found in the farm F and the *Phormidiaceae* were found in all farms. *Cyanobacteria* families were not evidenced at the beginning of the experiment; those bacteria are known as nitrogen-fixing bacteria. They are able to thrive in hostile environments as dryland areas and are pioneers in many nutrient-poor substrates (Hakkoum et al., 2021).

In the farms F and P, sediment drying had significantly reduced the NO_3_^−^ concentration and total sulfur compared to the beginning of the experiment; while in farm D, sediment drying has considerably increased the NO_3_^−^ and sulfur concentrations compared to the beginning of the experiment. For farm A the reduction of NO_3_^−^ levels was much lower. It would therefore appear that sediment drying did not have the same effect on the biogeochemical cycle, depending on the farm sediments (Table 1). In dry conditions, the sediment pH of the farm D was still very different from the others farms (Table 1). Furthermore, in dry sediment, the pH difference between farm D sediment and others farms was greater than at D0. The lower pH values of farm D could therefore explain the significant differences observed in the sediment microbiota of this farm compared to the others (Table 3) as pH was also reported as a main driving factor structuring soil bacterial community (Zhalnina et al., 2015; Tripathi et al., 2018; Wang et al., 2019). The initial composition of the microbial communities may have had a key role in sediment biogeochemical cycles (C, N, S). Indeed, the farms F and P for which the sediment microbiotas were similar at D0 (Figure 2C), had the same range of NO_3_^−^ and NH_4_^+^, total phosphorus and sulfur concentration in their dry sediment compared to the others farms (Table 1).

In dry sediments, farm D was distinguished from the others by a significantly lower relative abundance of *Comamonadaceae*, whereas this family was among the abundant in dry sediments from the other farms (Figure 2D). Specifically, members of this family related to the *Acidovorax*, *Brachymonas, Comamonas, Hydrogenophaga* genera, were reported to be able to denitrify and use nitrates (Willems, 2014). In several studies, the denitrifying bacteria found were mainly members of the *Comamonadaceae* family (Khan et al., 2002; Wang and Chu, 2016; Chu and Wang, 2017). Thus, occurrence of these family may have favored the reduction of nitrogen level in farms A, F and P compared to the beginning of the experiment. In farm A, we also found *Nitrosomonadaceae*, which are ammonia oxidizers, with some genera able to oxidize ammonia to nitrite and other genera to oxidizing nitrite to nitrate (Prosser et al., 2014). Thus, the occurrence of this family may explain the higher NO_3_^−^ concentration in the dry sediment of the farm A compared to farms F and P. However, this family was nearly absent in the dry sediment of the farms D, F, and P (Figure 2C). It may also be possible that some soil biogeochemical processes of denitrification, nitrification, sulfur oxidation and reduction may have occurred largely before our sampling time as we used RNA, which has a shorter lifespan and higher turn-over in the environment than the DNA molecule. Thus, some prokaryotes may not be identified because they were no more “active” in the dry sediment after 6 months (Cristescu, 2019; Wood et al., 2020). Indeed, we also did not evidenced prokaryotes linked to sulfur cycle in dry sediment while sulfur level was reduced in farms A, F and P. After 6 months of drying and based on the sediment microbiota solely, we also could not explain the increase of sulfur and NO_3_^−^ concentrations in farm D sediment.

### 4.3. Focus on *Suaeda australis* nutrient removal efficiency between farms sediment

#### 4.3.1. Specific microbiotas composition and putative functions

The growth of *Suaeda australis* in sediment had two opposite effects on alpha diversity according to the farms. In farms A and D, the alpha diversity index had increased compared to D0 whereas it decreased in farms F and P while they had the highest alpha diversity indices at the start of the experiment (Table 2). A previous study has also reported that cottons roots have different effect on alpha diversity of the rhizosphere bacterial community in two types of soils (Qiao et al., 2017). They showed that in nutrient-rich soils, bacterial diversity in the rhizosphere was lower, because some bulk soil microorganisms were unable to adapt to variations in chemical and physical properties of the soil and to adapt to variation of root exudates. In our study, the decrease in alpha diversity may reflect the plant’s selection of microorganisms from the bulk soil into its rhizosphere. In contrast, in sediments with lower alpha diversity (farms A and D), the release of root exudate may have enhanced the diversity of microorganisms.

The *Alphaprotebacteria* dominated the specific rhizosphere microbiota of *Suaeda australis* (Figure 5C). Bacteria of this class have the ability to interact with plants as pathogens, symbionts or non-symbionts (Pini et al., 2011). Other studies have also found that *Alphaproteobacteria* was the dominant class in the rhizosphere soil (Jorquera et al., 2016). Thus, *Suaeda australis* may had promoted the occurrence of *Alphaproteobacteria* in the rhizosphere. However, at the family level, *Suaeda australis* rhizosphere specific microbial communities’ composition and abundance were varying according to the farms (Figure 3B). For instance, farm D was marked by the predominance of nitrogen-fixing *Terasakiellaceae* (*Alphaproteobacteria* class) which was absent in others farms sediment (Figure 3B; Weiler et al., 2018; Espín et al., 2021). In the farms A and F, the rhizosphere microbiota *of Suaeda australis* had higher relative abundance of *Rhodobacteraceae* family (*Alphaproteobacteria* class) compared to the farms D and P. The *Rhodobacteraceae* family was also reported to be deeply involved in sulfur and carbon biogeochemical cycles (Pujalte et al., 2014). The higher occurrence of this family in the farms A and F may have favored the reduction of sulfur level in sediment compared to D0 (Table 1). A previous study has reported that members of *Rhodobacteraceae* family are key player in hydrocarbon degradation in Mexico beach sand (Kostka et al., 2011). Thus, higher abundance of taxa from this family in farm A may explained its significant link to hydrocarbon degradation function (Figure 4).

Functions linked to biopolymer lysis were found significantly enriched in the farms F and P with celluloysis and chitinolysis functions. The chitinolysis function is relevant because chitin makes up the shrimp exoskeleton and this biopolymer accumulates in the sediments during shrimp rearing as the shrimps molt or die (Philip and Antony, 2006; Della Patrona and Brun, 2009). Those functions may be linked to the higher abundance of several families related to *Myxobacteria* (*Haliangiaceae, Nannocystaceae, Sandaracinaceae*) in farms F and P (Mohr, 2018). *Myxobacteria* were also found in farms A and D but in a lesser abundance. However, the occurrence of *Myxobacteria* in the specific rhizosphere microbiota may not be due to the plant influence as those families were found without plant occurrence on D0 and in dry sediment.

In farm F, the specific microbiota of *Suaeda australis* was significantly correlated with pathogens functions (human pathogens, animal parasites or symbionts) which may be explained by the presence of *Alphaproteobacteria* from the *Sphingomonadaceae* family. In fact, some genera of *Sphingomonadaceae* are known as plant pathogens or to cause human infection (Glaeser and Kämpfer, 2014). The functional assignment of prokaryotic taxa with FAPROTAX is based on information on taxa using standard references (Louca et al., 2016a,b). The FAPROTAX tool is relevant for predicting the functions of prokaryotes related to biogeochemical cycles. It can be applied to the analysis of the microbiome (terrestrial, human, animal), however, it is not specialized in the microbiome of shrimp sediment (Sansupa et al., 2021).

The *Desulfuromonadia* class was evidenced in the rhizosphere of *Suaeda australis* in farms A and P. It belongs to the *Desulfurobacterota* phylum that encompasses sulfate-reducing and fermentative taxa (Murphy et al., 2021). However, the abundance of the families affiliated to this phylum varied within farms, with *Geothermobacteraceae* and *Bradymonadaceae* found only in farms P and A, respectively. This evidenced that sediment types had influence the structuration of rhizosphere microbial composition. Previous study has also reported differences in the composition of the rhizosphere microbial communities of *Arabidopsis thaliana*, lettuces plants or also cotton grown in different types of terrestrial soil (Lundberg et al., 2012; Schreiter et al., 2014; Qiao et al., 2017). In their study, Qiao et al. (2017) reported that the soil background microorganisms were the main cause of the variation of the microbial community in the rhizosphere between different soils. We have highlighted in this study, that at the beginning of the experiment, the microbial diversity and composition varied between the four farms. Thus, as the rhizosphere communities were a subset of soil microbial communities, differences in the initial pool of soil prokaryotic taxa between farms may result in different recruitment of taxa by *Suaeda australis*. Also, variation of rhizosphere microbial communities between farms may also result from the growth and nutrition of *Suaeda australis* that could influence the chemical parameters of the sediments. Indeed, we have evidenced that under *Suaeda australis* cultivation, the pH, nitrogen and sulfur concentrations varied in different ways between the farms (Table 1). For instance, Marschner et al. (2004), evidenced that variation of soil pH value of one unit, can significantly affect the structure of the bacterial communities of Sudan grass rhizosphere, grown in sandy loam soil. In addition, variations in the amount of root exudation according to the farms sediments could also be a factor that influencing the microbial composition of the *Suaeda australis* rhizosphere (Neumann et al., 2014; Maurer et al., 2021).

#### 4.3.2. Evaluation of the sediment nutrient removal efficiency between farms

Cultivation of *Suaeda australis* in the sediments seemed effective in removing nitrogen, sulfur and phosphorus but this varied according to the farms (Table 1). The nitrogen concentration was reduced in farms A, F and P whereas the total sulfur was reduced only in farms A and F. For all the farms, the cultivation of *Suaeda australis* has reduced both the total and available forms of phosphorus (Table 1). In all farms, the cultivation of *Suaeda australis* had increased the concentration of organic carbon in the sediment compared to D0 (Table 1). This increase may be due to plant litter or to the exudates released by the roots (Yan et al., 2018).

The sediment of the farm D was the only one for which the NO_3_^−^ level had increased significantly under both *Suaeda australis* cultivation and sediment drying compared to D0 (Table 1). In contrast, in other sediments from shrimp farms, nitrogen levels were reduced by 55–99% under *Suaeda australis* cultivation. In addition, for farm D, the practice of drying or growing halophytes increased total sulfur and organic carbon concentrations by at least 36 and 40% respectively, which was significantly higher than the concentrations observed in the others farms (Table 1). Thus, theses experimental conditions have selected microbial communities that appeared to be not effective for sediment nitrogen, carbon and sulfur removal. Farm D also stood out from the others for its lower pH values, higher ammonia concentration and lower alpha diversity index (Tables 1, 2). The lower pH value observed in farm D at the initial state and in dry conditions compared to the other farms may have not favor nutrient removal by rhizosphere microbial communities. It was reported that soil acidity reduces the intrinsic activities of microbial communities (Kemmitt et al., 2006), this could explain the significantly increased of organic carbon and nitrogen in the dry sediment and with the halophytes (Table 1). Under the cultivation of *Suaeda australis* the pH of the sediment of the farm D increased but it was still lower than the other farms (Table 1). Thus for farm D, liming the sediment accumulation beforehand halophyte cultivation or drying period may be a way to increase the sediment pH and to improve the organic matter decomposition by microbial communities (Li et al., 2015). In addition to control sediment acidity, the liming application in shrimp sediment can also be beneficial to improve the sediment texture as the porosity (Della Patrona and Brun, 2009). This sediment parameter could also shape the microbial diversity and composition (Chau et al., 2011; Seaton et al., 2020).

For the other farms, sediment drying appears more efficient than *Suaeda australis* cultivation for the reduction of total sulfur. This can be explained by the fact that in the greenhouse, pots containing halophytes were watered twice a week with seawater, which is a source of sulfate for the sedimentary matrix. In addition, plant residues can be sources of organic sulfur for the sediments (Schoenau and Malhi, 2015). It would be therefore interesting to determine the different forms of sulfur in the sediments, such as the quantity of sulfur in its organic form that is available to the plant, or the quantity of bound sulfur, for a better understanding of the effect of halophyte cultivation on the sulfur cycle (Zhou et al., 2009). For all the farms, the available forms of phosphorus were reduced almost as much in the dry sediment as in the halophyte rhizosphere. This may be explained as phosphorus is an essential nutrient for microorganisms growth (Oliverio et al., 2020) and therefore microbial communities have used the pool of available sediment phosphorus for their nutrient requirement. This highlighted a pivotal role of the prokaryotes in the phosphorus cycle.

In the farm A, the nitrogen removal under the halophyte cultivation was significantly higher than in the dry sediment. Thus, for this farm, *Suaeda australis* nutrition and its associated microbiota was more effective than in dry condition to reduce nitrogen level accumulated in the sediment. However, in the farms F and P, the practice of drying alone, had greatly reduced the level of nitrogen in the sediments (Table 1), which meant that for these two farms, the microbial community alone was almost as effective (farm F) or even more effective (farm P) than the cultivation of halophytes and its microbiota. Those two farms had similar microbial communities at the beginning of the experiment, higher alpha diversity indices and higher CaCO_3_ concentration in their sediments (Figure 2C and Tables 1, 2). Those parameters may have favored a better nutrients removal in the sediment with and without halophyte cultivation. However, for those two farms F and P, the reduction of the total phosphorus was significantly higher under halophyte cultivation compared to dry sediment. This higher decrease under *Suaeda australis* cultivation may be linked to the phosphorus nutrition for the plant. In the soil, mineralization of organic P is catalyzed by hydrolytic enzymes such as phosphatase that can be released by plant or soil microorganisms (Marschner et al., 2011; Nannipieri et al., 2011). The mineralization of organic phosphorus is thereby controlled by the plant and microbial demand (Richardson and Simpson, 2011). Thus, a higher mineralization of sediment phosphorus to meet the plant needs could have explained the greater reduction of total phosphorus in the *Suaeda australis* rhizosphere compared to the dry sediment. This could be the case particularly for the farm F, where the total pool of phosphorus available to the plant was significantly lower than for all the other farms (Table 1). Thus, the effectiveness of nutrient removal by halophyte in shrimp sediment seemed to be strongly influenced by the chemical characteristics of the sediments at the initial state. The aquaculture sediments are formed on the initial soil of the pond as soon as aquaculture activities begin, and then evolve with successive rearing and zootechnical practices (Munsiri et al., 1995). It was also reported that in New Caledonia, the pond sediment pH and CaCO_3_ concentration may change with the aging of farms (Lemonnier et al., 2021). Thus, the background of farms aquaculture practices, farms age or type of original pond soil may explain variation of sediment chemical parameters between farms.

## 5. Conclusion

First, our results showed that the active microbial diversity of sediments varied between shrimp farms, which has so far never been explored before in New Caledonia. The initial CaCO_3_ concentration in the sediments appeared to have significantly influenced the microbial diversity, with the higher alpha diversity indices and microbiota similarities found in sediments with the higher CaCO_3_ concentrations.

The difference in microbial and chemical composition between the farms led to different specific microbiota in the *Suaeda australis* rhizosphere, recruited from the bulk sediments, as well as in the selection of various putative microbial function in the sediment. *Alphaproteobacteria* class was promoted in *Suaeda australis* rhizosphere but the related family composition and abundance varied according to the farm sediments. Thus, the initial microbial composition and chemical characteristic of the sediment seemed to have strongly influenced the efficiency of nutrient removal by *Suaeda australis*. In fact, the significant efficient nitrogen removal without halophyte cultivation in the farms F and P sediments meant that in those farms, the microbial communities played an important and efficient role in the nitrogen cycle. The pH had also emerged as a key factor in the success of nutrient removal and thus in nitrogen removal under halophyte cultivation or sediment drying. In addition, the sediment microbial community of this farm was more different from the other farms. Therefore, the initial chemical parameters of these aquaculture sediments should probably be improved to ensure better nutrient removal efficiency; liming may be a solution to improve pH, texture and neutralize acidity in the sediments.

In conclusion, this study demonstrated the impact of *Suaeda australis* cultivation on nitrogen, sulfur, and phosphorus removal in shrimp pond sediments; however, the removal efficiency varied depending on the sediment of the shrimp ponds. Thus, the initial chemical and microbial composition of the sediment must be studied in order to select the most appropriate method for nutrient removal (halophyte, drying, lime application, or a combination of several methods). To go further, it would be interesting to repeat this experiment but at the pond scale; and also to compare the nutrient removal efficiency among different halophyte species or a mixture of halophyte species within pond sediment. Thus, ultimately some halophyte species could be rather efficient for the nutrient removal of a type of sediment than another.

## Data availability statement

The datasets presented in this study can be found in online repositories. The names of the repository/repositories and accession number(s) can be found at: NCBI BioProject (https://www.ncbi.nlm.nih.gov/bioproject/), PRJNA996981.

## Author contributions

MC: Conceptualization, Investigation, Methodology, Software, Writing – original draft, Writing – review & editing, Data curation, Formal analysis, Visualization. LG: Supervision, Validation, Writing – review & editing. LD: Conceptualization, Funding acquisition, Investigation, Methodology, Supervision, Validation, Writing – review & editing. DA: Methodology, Writing – review & editing. NC: Data curation, Funding acquisition, Investigation, Software, Supervision, Validation, Visualization, Writing – review & editing, Formal analysis, Methodology.

## Footnotes

^1^ https://benjjneb.github.io/dada2/tutorial_1_6.html

^2^ http://jvenn.toulouse.inra.fr
