# Supplementary material for: Suaeda australis and its associated rhizosphere microbiota: a comparison of the nutrient removal potential between different shrimp farm sediments in New Caledonia
Source: Front Microbiol. 2023 Oct 9;14:1260585. doi: 10.3389/fmicb.2023.1260585 (PMC10591223; doi:10.3389/fmicb.2023.1260585)
Supplement: Supplementary file 1 [file Table_1.DOCX]

Supplementary Material

# Supplementary Tables

**Tab.1**. Abundance of bacteria families found in the sediments microbiota at the start of the experiment (D0) in the different shrimp farms (A, D, F and P).

| Family | A | D | F | P |
| --- | --- | --- | --- | --- |
| Alteromonadaceae | 0.18 | 1.32 | 0.86 | 2.02 |
| Bacillaceae | 0.76 | 2.08 | 0.22 | 0.28 |
| Balneolaceae | 6.63 | 1.86 | 1.47 | 2.70 |
| Bradymonadaceae | 1.67 | 1.51 | 0.14 | 0.23 |
| Cellvibrionaceae | 0.19 | 0.72 | 8.65 | 0.73 |
| Chromatiaceae | 3.27 | 2.40 | 0.28 | 0.31 |
| Cyclobacteriaceae | 1.43 | 1.93 | 1.68 | 1.13 |
| Flavobacteriaceae | 4.11 | 17.56 | 8.01 | 10.80 |
| Halomonadaceae | 1.81 | 0.96 | 1.31 | 3.34 |
| Idiomarinaceae | 2.78 | 4.94 | 1.74 | 0.17 |
| Marinobacteraceae | 3.77 | 2.85 | 3.56 | 3.68 |
| Nannocystaceae | 0.07 | 0.03 | 1.04 | 5.92 |
| Nitriliruptoraceae | 0.79 | 2.42 | 1.49 | 1.00 |
| Nitrosomonadaceae | 0.85 | 0.08 | 0.52 | 1.72 |
| Rhodobacteraceae | 3.91 | 4.62 | 4.28 | 4.47 |
| Rhodothermaceae | 2.87 | 4.19 | 3.39 | 3.40 |
| Saccharospirillaceae | 0.29 | 0.40 | 1.27 | 0.90 |
| Sandaracinaceae | 0.45 | 0.12 | 8.14 | 9.90 |
| Thioalkalibacteraceae | 22.68 | 21.80 | 0.18 | 0.27 |

**Tab.2.** Abundance of bacteria families found in the dry sediments microbiota at the end of the experiment (6 months) in the different shrimp farms (A, D, F and P).

| Family | A | D | F | P |
| --- | --- | --- | --- | --- |
| BIrii41 | 2.2 | 1.0 | 0.2 | 1.0 |
| Bryobacteraceae | 2.2 | 0.2 | 0.5 | 2.5 |
| Caulobacteraceae | 0.7 | 3.4 | 0.1 | 0.5 |
| Comamonadaceae | 7.3 | 1.0 | 5.3 | 17.5 |
| Cyclobacteriaceae | 0.4 | 6.4 | 0.7 | 0.3 |
| Gemmatimonadaceae | 2.9 | 0.6 | 2.0 | 3.0 |
| Haliangiaceae | 5.9 | 4.7 | 1.0 | 1.3 |
| Leptolyngbyaceae | 0.0 | 0.0 | 5.3 | 0.1 |
| Longimicrobiaceae | 6.3 | 2.5 | 1.5 | 6.4 |
| Myxococcaceae | 3.2 | 2.2 | 1.4 | 14.5 |
| Nannocystaceae | 0.1 | 0.4 | 5.3 | 0.1 |
| Nitrosomonadaceae | 3.3 | 0.2 | 0.4 | 1.0 |
| Nostocaceae | 0.2 | 0.1 | 12.1 | 7.6 |
| Phormidiaceae | 5.8 | 2.9 | 2.9 | 2.0 |
| Polyangiaceae | 6,8 | 9,9 | 0,7 | 0,9 |
| Rhodobacteraceae | 2,6 | 3,3 | 5,5 | 1,0 |
| Rhodothermaceae | 0,7 | 3,8 | 1,4 | 0,6 |
| Sandaracinaceae | 1,0 | 1,6 | 2,0 | 1,7 |
| Sphingomonadaceae | 3,0 | 2,2 | 1,1 | 0,9 |

**Tab.3**. Abundance of bacteria families found in the specific microbiota of the *Suaeda australis* at the end of the experiment (6 months) in the different shrimp farms (A, D, F and P).

| Family | A | D | F | P |
| --- | --- | --- | --- | --- |
| A4b | 0.8 | 0.7 | 5.7 | 2.0 |
| Anaerolineaceae | 0.2 | 0.9 | 0.0 | 1.5 |
| Balneolaceae | 10.8 | 3.6 | 0.0 | 0.3 |
| BIrii41 | 1.5 | 1.4 | 1.3 | 1.7 |
| Bradymonadaceae | 3.0 | 0.0 | 0.0 | 0.0 |
| Cyclobacteriaceae | 0.9 | 3.5 | 0.5 | 0.5 |
| Desulfuromonadaceae | 0.0 | 0.0 | 0.6 | 0.0 |
| Flavobacteriaceae | 0.2 | 3.3 | 0.3 | 0.0 |
| Geothermobacteraceae | 0.1 | 0.0 | 0.0 | 5.8 |
| Haliangiaceae | 0.5 | 1.5 | 5.9 | 0.1 |
| Micromonosporaceae | 5.3 | 1.3 | 0.0 | 0.2 |
| Microscillaceae | 0.0 | 0.0 | 0.6 | 0.0 |
| Nannocystaceae | 1.0 | 1.0 | 4.4 | 5.7 |
| Nitriliruptoraceae | 0.0 | 1.3 | 0.0 | 0.2 |
| Nitrosomonadaceae | 0.1 | 0.0 | 2.2 | 0.0 |
| Nodosilineaceae | 2.1 | 0.0 | 0.6 | 8.0 |
| Nostocaceae | 1.5 | 0.0 | 2.6 | 0.5 |
| NS11-12 marine group | 0.8 | 0.0 | 0.3 | 0.2 |
| Oscillatoriaceae | 0.0 | 0.0 | 7.3 | 0.0 |
| Rhodobacteraceae | 12.3 | 2.5 | 11.0 | 6.0 |
| Rhodothermaceae | 0.3 | 1.2 | 0.1 | 0.7 |
| Sandaracinaceae | 1.1 | 1.1 | 2.8 | 1.2 |
| Sphingomonadaceae | 0.3 | 0.9 | 3.3 | 0.0 |
| Terasakiellaceae | 0.6 | 36.5 | 0.0 | 0.0 |
| TRA3-20 | 0.0 | 0.0 | 1.6 | 0.0 |
